# Central nervous system involvement in relapsing polychondritis with inflammatory changes in the cerebrospinal fluid and cranial MRI abnormalities: a case series report

**DOI:** 10.3389/fmed.2026.1962270

**Published:** 2026-10-30

**Authors:** Jie Chen, Zhewen Luo, Ping Hua, Biying Wang, Xing Wang

**Affiliations:** 1 Department of Neurology, The Affiliated Brain Hospital of Nanjing Medical University, Nanjing, China; 2 Affiliated Hospital of Integrated Traditional Chinese and Western Medicine, Nanjing University of Chinese Medicine, Nanjing, China; 3 School of Integrated Chinese and Western Medicine, Jiangsu Health Vocational College, Nanjing, China

**Keywords:** central nervous system involvement, clinical manifestations, inflammatory changes, MRI features, relapsing polychondritis

## Abstract

**Objective:**

To explore the clinical features of relapsing polychondritis (RP) with central nervous system (CNS) involvement as the major manifestation.

**Methods:**

A total of 11 RP patients who were hospitalized in the Department of Neurology, the Affiliated Brain Hospital of Nanjing Medical University from January 2020 to December 2024 were retrospectively enrolled. Their clinical manifestations, laboratory indicators, cerebrospinal fluid (CSF) changes, and cranial magnetic resonance imaging (MRI) findings were comprehensively analyzed.

**Results:**

CNS involvement was the major initial manifestation in the vast majority of RP patients (90.9%, 10/11), as cognitive impairment (*n* = 10), headache (*n* = 7), psychiatric/behavioral symptoms (*n* = 6), epileptic seizures (*n* = 4), limb numbness (*n* = 4), limb weakness (*n* = 4), and limb tremor (*n* = 3). CSF analysis revealed an inflammatory response in the CNS, as evidenced by increased levels of neutrophils, albumin, and immunoglobulins at varying degrees. White matter degeneration (*n* = 7) and brain atrophy (*n* = 7) were the pronounced changes on cranial MRI. Brain lesions were mainly distributed in the cortex, subcortex, and infratentorium. Parenchymal or meningeal enhancement was observed on MRI scans in three RP patients with CNS involvement. Arterial spin labeling (ASL) detected three cases of hypoperfusion and two cases of hyperperfusion in the brain. All patients were treated with corticosteroids, and six were managed with additional immunotherapies. Finally, ten RP patients were improved and discharged.

**Conclusion:**

neurological deficits vary widely in RP patients with CNS involvement, with cognitive decline as the most common feature. Inflammatory changes in the CSF indicate a neuroinflammatory mechanism involved in the pathogenesis of RP. Distinct imaging features on cranial MRI are closely associated with the involvement into CNS in RP, offering references for distinguishing the acute inflammation stage from the chronic degeneration stage during the development of RP.

## Introduction

Relapsing polychondritis (RP) is a rare, systemic, highly heterogeneous autoimmune disease that often invades body parts rich in cartilage (e.g., ears, nose, and trachea). It can also endanger the heart, kidneys, and central nervous system (CNS). Neurological deficits in RP are reported in only 11% of patients (1, 2). CNS involvement as the major manifestation of RP is even rarer and can be easily misdiagnosed (3).

In this case series report, we retrospectively analyzed the clinical data, laboratory indicators, cerebrospinal fluid (CSF) changes, and cranial magnetic resonance imaging (MRI) features among 11 RP patients with CNS involvement. Through a comprehensive analysis on CNS involvement in RP, our findings are expected to provide references for increasing diagnostic accuracy and improving patients’ prognosis.

## Methods

### Study population

Consecutive RP patients with CNS involvement who were hospitalized in the Department of Neurology, the Affiliated Brain Hospital of Nanjing Medical University from January 2020 to December 2024 were retrospectively enrolled in the present study, involving 7 (63.6%) male and 4 (36.4%) female patients at a mean age of 62.7 ± 9.3 (47–79) years. Their clinical manifestations, laboratory indicators, cerebrospinal fluid (CSF) changes(OCB and Reiber intrathecal IgG synthesis analysis were not routinely performed) and cranial magnetic resonance imaging (MRI) findings were comprehensively analyzed. This was a retrospective case series study, and all personally identifiable information had been thoroughly desensitized. Clinical photos had been analyzed under informed consent. [The study was approved by the Ethics Committee of the Affiliated Brain Hospital of Nanjing Medical University, Approval No. (2026-KY055-01)].

### Inclusion and exclusion criteria

Inclusion criteria were set as the diagnostic criteria for RP modified by Damiani et al. (4) and Michet et al. (5). CNS involvement was comprehensively determined by typical clinical symptoms and signs (e.g., headache, cognitive impairment, psychiatric/behavioral symptoms, epileptic seizures, numbness and weakness of limbs, tremor, and ataxia) and positive findings in CSF analysis (increased white blood cell counts or protein levels that indicated inflammatory responses), electroencephalogram (EEG), cranial MRI neuroimaging, electromyogram (EMG), and vestibular function testing, ruling out the cause of other diseases.

Exclusion criteria are as follows:

Neuroinflammatory: CNS infection, multiple sclerosis, PACNS, and other autoimmune neuropathies; Cerebrovascular diseases: acute stroke attack and cerebral small vessel disease; Neurodegenerative diseases: Alzheimer's disease.
Diagnostic rationale: All included patients satisfied the inclusion criteria and were free of exclusion criteria. Besides that, all patients developed neurological signs and symptoms during the course of RP rather than as independent events, and they did not exhibit typical features of other neuroimmunological and inflammatory diseases (multiple sclerosis, polyneuropathy, primary angiitis of the central nervous system). Furthermore, the neurological inflammatory and imaging findings were inconsistent with the diagnostic criteria for the above neuroimmunoinflammatory diseases, as explained below: (i) Multiple sclerosis was excluded because no typical spinal cord lesions on MRI. (ii) Primary angiitis of the CNS (PACNS) was excluded on the basis of the MRI/MR venography findings, the negative systemic vasculitis work-up, and the clinical course. (iii) Other inflammatory/autoimmune neuropathies were excluded by neurological examination and, where available, nerve conduction studies/EMG, together with the negative paraneoplastic and autoimmune antibody panels (Figure 1).

**Figure 1:**
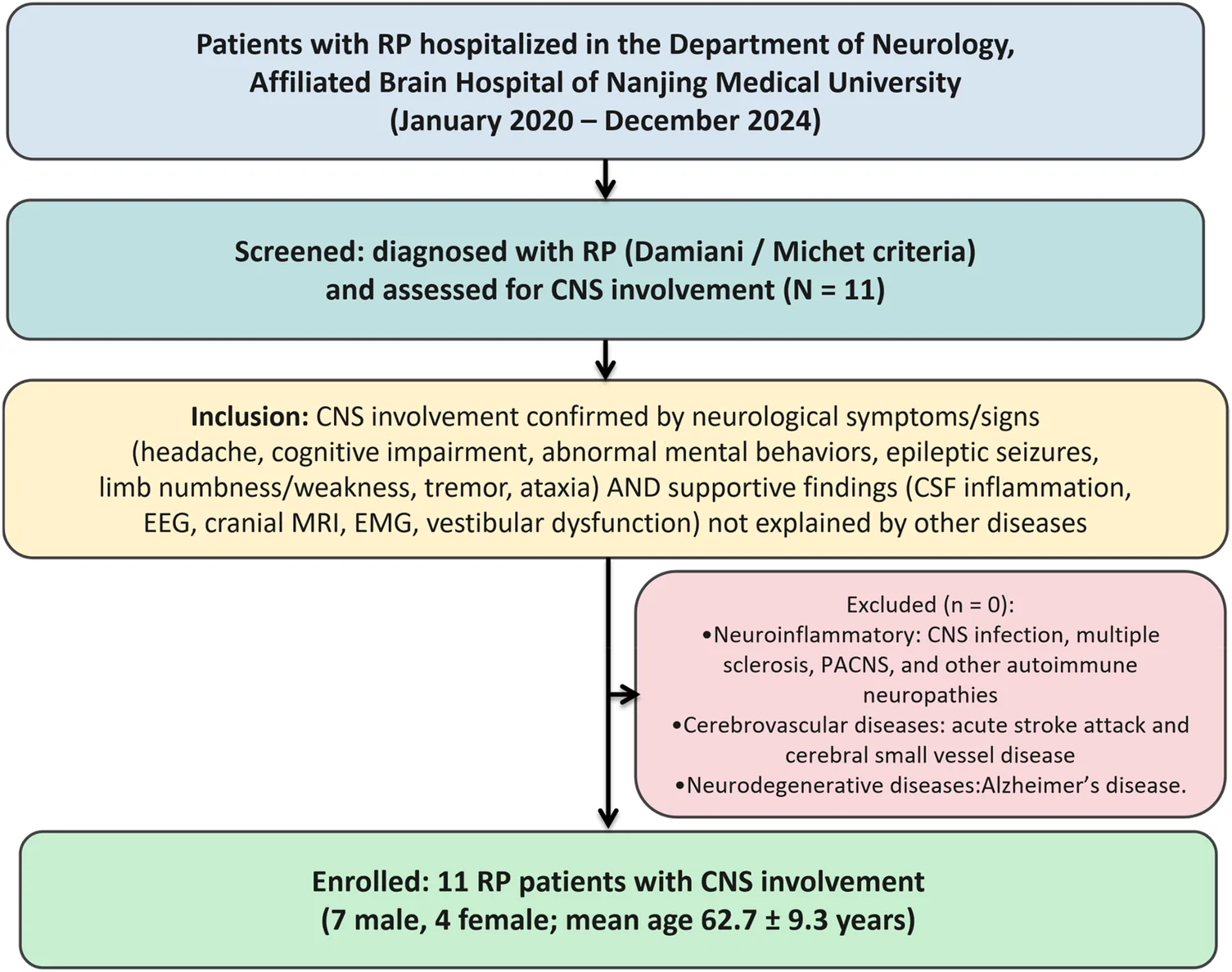
Flowchart of patient enrollment, inclusion, and exclusion.

### Imaging assessment

Cranial MRI scans, including plain MRI, contrast-enhanced MRI, and arterial spin labeling (ASL) sequences. White matter degeneration was defined as diffuse or confluent hyperintense lesions on T2-weighted or fluid-attenuated inversion recovery (FLAIR) images, and brain atrophy was defined as diffuse or localized reduction of brain parenchymal volume accompanied by widening of the sulci and ventricles. Focal parenchymal lesions, contrast enhancement, and ASL perfusion abnormalities were recorded according to their location and pattern.

All imaging examinations are first formally reported by a neuroradiologist. Given the rarity of RP-CNS involvement, the neuroradiological reports were then independently reviewed by two neurologists with expertise in neuroimaging, who analyzed the imaging features in a blinded fashion. Finally, the neurologists discussed the findings with the neuroradiologist, and a consensus conclusion was reached through this multidisciplinary discussion. When consensus could not be reached, the final judgment was made by a third senior neurologist.

### Data collection

Outpatient and inpatient medical records were collected from all enrolled RP patients for analyzing their demographic data, clinical manifestations, laboratory indicators, imaging findings, treatment regimens, outcomes, etc.

### Statistical analysis

SPSS 25.0 was used for statistical processing. Enumeration data were expressed as the number of cases and corresponding percentage. Measurement data within a normal distribution were expressed as mean ± standard deviation $\left(\bar{x}\pm s\right)$; otherwise, they were denoted as the median and interquartile range (IQR).

## Results

### Clinical manifestations of CNS involvement in RP

In our retrospective cohort, the diagnosis of CNS involvement in RP was often delayed, ranging from 1 to 15 months from the initial onset of neurological symptoms to a definite diagnosis, with a mean duration of 4.4 ± 4.5 months and a median value of 2 months. Specifically, a delayed diagnosis of CNS involvement in RP within one month was found in nearly half of the RP patients (45.5%, 5/11). The diagnosis was delayed by 2, 5, 8, and even 15 months in 2 (18.2%), 2 (18.2%), 2 (18.2%), and 1 (9.1%) case of RP, respectively. Among the 11 patients, 5 patients (45.5%) presented with the recurrence or worsening of CNS manifestations. Cognitive impairment (90.9%, 10/11) was the most common manifestation of neurological deficits in RP patients with CNS involvement, followed by headache (63.6%, 7/11) and psychiatric/behavioral symptoms (54.5%, 6/11). Other manifestations of neurological deficits consisted of movement abnormalities, epileptic seizures, limb tremor, ataxia, and urinary retention (Table 1).

**Table 1:** Consolidated clinical characteristics of 11 patients with relapsing polychondritis and central nervous system involvement.

Demographics & diseasecourse						Extra-CNSinvolvement	Neurologicalmanifestations	Laboratory findings					CSFassessment	EEGexamination	Imaging	Treatment & outcome		
Case	Age	Sex	Initial symptoms	Diagnostic delay	Combined risk factors for cerebrovascular disease	Extra-CNS organ involvement	Neurological manifestations	ESR	CRP	RF	Auto-antibodies	AE/paraneo-plastic antibodies	ICP/cells/protein/IgG	EEG/Video EEG	Cranial MRI	Treatment	Outcome at discharge	Follow-up outcome
	(years)			(months)	(years)			(mm/h)	(mg/L)	(IU/mL)			(mmH₂O/μL/g/L/mg/L)					
1	68	F	Fever, headache, auricular redness and swelling	8	None	Auricular redness and swelling; fever; right ear redness.	Epileptic seizures, hallucinations, memory decline, euphoria, slowed response, behavioral disturbance, urinary retention.	N/A	14.11	N/A	Negative	Negative	175/62/1.35/261	Moderate abnormal EEG with focal abnormalities; sphenoidal electrode EEG showed generalized moderate abnormality with localized abnormalities.	Bilateral hippocampal atrophy, white matter degeneration.	Antiviral therapy + dexamethasone 10 mg for 7 days + leflunomide 10 mg q.d.	Occasionally failed to recognize family members; memory decline; discharged on oral prednisone 30 mg q.d.	Relapse
2	58	F	Headache	5	None	Auricular pain and swelling; redness and pain of the fingers and knees; hearing loss; conjunctivitis.	Headache, increased muscle tone, decreased calculation ability, neck stiffness, bilateral lower limb tremor.	36	41.67	343.4	Negative	Negative	280/176/1.66/222	Generalized moderate-to-severe abnormal EEG.	Chronic hypoxic white matter changes, brain atrophy.	Dexamethasone 10 mg for 3 weeks + azathioprine 50 mg q.d.	Headache and dizziness improved.	
3	63	M	Red eye	1	Hypertension (8)	Auricular redness and swelling, joint redness and swelling, rash, low-grade fever, conjunctivitis.	Numbness and weakness of the four limbs, memory decline, glove-and-stocking hypoesthesia.	58	115.69	Normal	Negative	Negative	120/6/0.71/126	Generalized mild abnormal EEG with generalized alpha activity.	Contrast-enhanced MRI: multiple abnormal enhancements in basal ganglia, thalamus, and brainstem; MRV: multiple abnormal signals; ASL: increased perfusion signal adjacent to the right lateral ventricle.	Methylprednisolone 500 mg for 5 days with half-dose tapering + intravenous cyclophosphamide.	Auricular redness and swelling subsided, limb weakness recovered.	
4	69	M	Headache	1	Hypertension (21);Diabetes (12).	Ear pain	Headache	100	41.62	Normal	Negative	NGS performed	105/14/0.51/66.8	Normal EEG.	Mild cerebral atherosclerosis.	Methylprednisolone 1 g for 3 days, then 500 mg for 5 days, tapered over 3 days.	Headache improved.	Partial improvement with persistent deficits
5	75	F	Fever, headache	1	Hypertension (19).	Fever, bilateral hearing loss, recurrent auricular redness and swelling for over 1 year.	Headache, slowed response, psychiatric symptoms, dizziness, unsteady gait.	40	10.52	Normal	Negative	NGS performed	120/60/0.66/143	Generalized moderate abnormal EEG.	Leptomeningeal enhancement.	Methylprednisolone 250 mg with a 7-day taper + Tripterygium wilfordii + hydroxychloroquine.	Headache, dizziness, and unsteady gait improved.	
6	56	M	Headache	8	None	Fever, swelling of the finger joints of the four limbs, right auricular redness and swelling with herpes.	Headache, hallucinations, tremor of the four limbs, slowed response, unsteady gait, numbness of the four limbs, impaired consciousness, neck stiffness, epileptic seizures.	26	24.73	Normal	Anti-Jo-1 (indeterminate)	Negative	Not cooperated with CSF analysis.	Not documented	Contrast-enhanced scan showed obvious linear leptomeningeal enhancement in the sulci.	Antiviral therapy + corticosteroid (methylprednisolone 200 mg for 3 days, discontinued due to gastrointestinal bleeding).	Treatment discontinued due to gastrointestinal bleeding.	Persistent neurological deficits
7	57	M	Bilateral lower limb weakness, slowness of movement, low-grade fever, headache	1	Hypertension (8);diabetes (7)	Auricular redness and swelling; history of swelling and pain of multiple joints; bilateral hearing loss.	Bilateral lower limb weakness, slowness of movement, fever, headache, slowed response, difficulty in verbal communication, neck stiffness.	62	33.98	Normal	Negative	Negative	95/35/2.6/264	Generalized mild-to-moderate abnormal EEG.	Abnormal white matter signals, brain atrophy; slight abnormality on MR spectroscopy.	Methylprednisolone 80 mg for 2 weeks.	Headache relieved, still with slowed response.	Partial improvement with persistent deficits
8	57	F	Conjunctivitis, auricular redness and swelling, joint swelling and pain	2	None	History of bilateral auricular redness and swelling in 2021; conjunctival congestion.	Slowed response, speech disturbance, behavioral abnormality.	70	16.72	Normal	Negative	Negative	65/19/0.6/74.2	Generalized moderate abnormal EEG.	Abnormal signals in the white matter of both cerebral hemispheres (white matter lesions).	Pulse corticosteroid (methylprednisolone 500 mg for 5 days) + mycophenolate mofetil.	Psychiatric behavior improved, still with cognitive impairment.	
9	79	M	Slowed response	5	Diabetes (18)	Redness and swelling of the bilateral auricular cartilage	Behavioral abnormality, slowed response, epileptic seizures, impaired consciousness.	39	116.53	Normal	Negative	Negative	40/46/0.6/69.1	Video EEG: focal abnormal EEG.	Multiple lacunar infarctions, white matter degeneration.	Pulse corticosteroid (methylprednisolone 500 mg for 4 days).	Still with slowed response, no response to questions, self-talking.	
10	47	M	Not recorded	1	Diabetes (3)	None	Headache, fever, epileptic seizures, increased muscle tone of the right upper limb, bilateral ankle clonus.	27	14.15	N/A	N/A	N/A	195/22/0.94/91.3	Sleep EEG showed no abnormality.	Lesions in the right temporal lobe with the bilateral hippocampus and white matter.	Methylprednisolone 80 mg for 1 week; cyclophosphamide 0.4 g once weekly recommended.	Slowed response and unable to answer.	
11	61	M	Hearing loss, dizziness	15	Hypertension (10)	Bilateral auricular Redness; conjunctival congestion; redness, swelling, and pain of the third metacarpophalangeal joints of both hands; tinnitus.	Slowed response, numbness and weakness of the four limbs, psychiatric behavioral abnormality.	4	Normal	Normal	Negative	Negative	ICP not detected/12/1.9/149	Generalized mild abnormal EEG.	Subcortical abnormal signals in the bilateral frontoparietal lobes (possibly demyelinating changes); bilateral hippocampal atrophy.	Methylprednisolone 60 mg for 3 days.	Still with slowed response and psychiatric behavioral abnormality.	

CSF findings are expressed as intracranial pressure (80–200 mmH_2_O)/white blood cell count (nucleated cell), (0–5/μL)/CSF total protein (0.15–0.45 g/L)/CSF immunoglobulin (0–34 mg/L).

CNS, central nervous system; RP, relapsing polychondritis; RF, rheumatoid factor; AE, Autoimmune Encephalitis; Relapse (indicating recurrence or worsening of CNS manifestations during follow-up); N.A., not applicable.

### Extra-CNS manifestations in RP

Besides CNS involvement, extra-CNS symptoms and signs of RP were also observed, including auricular chondritis (*n* = 10), joint swelling and pain (*n* = 6), conjunctivitis (*n* = 5), skin rashes (*n* = 2), and tracheal chondritis (*n* = 2). No one suffered from nasal cartilage symptoms of RP. These symptoms could be manifested either before (ranging from 2 months to 2 years prior to CNS involvement, *n* = 8), simultaneously (*n* = 1), or after (ranging from 5 months to 7 months after CNS involvement, *n* = 2) the occurrence of neurological deficits (Figure 2, Table 2).

**Figure 2:**
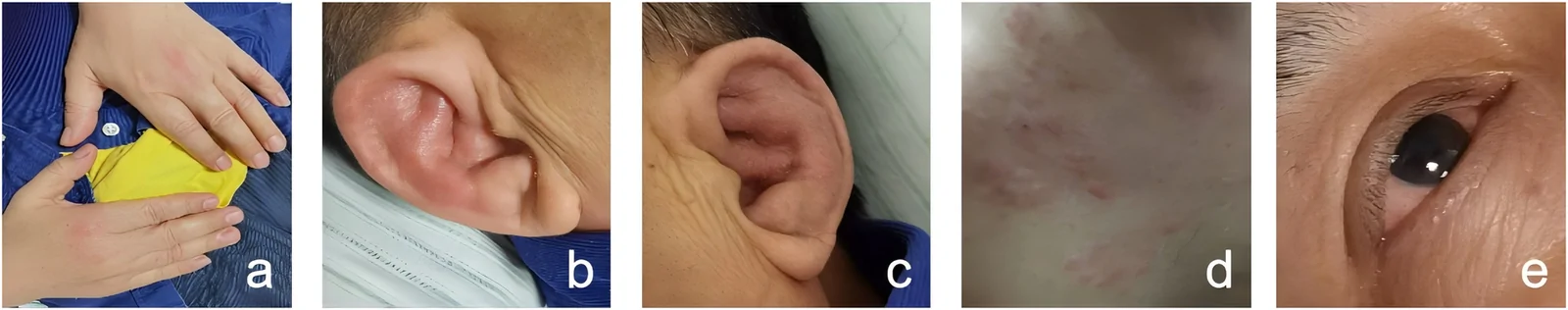
Representative images of extra-CNS manifestations in RP patients. **(a)** Finger joint swelling. **(b,c)** Auricular swelling with earlobe sparing. **(d)** Skin rashes on the anterior chest wall. **(e)** Conjunctivitis.

**Table 2:** Extra-CNS manifestations in RP.

Extra-CNS manifestations	*N* (%)
Fever	5 (45.5)
Auricular chondritis	10 (90.9)
Arthritis	6 (54.5)
Conjunctivitis	5 (45.5)
Vestibulocochlear symptoms	4 (36.4)
Inflammation in the tracheal cartilage	2 (18.2)
Skin rashes	2 (18.2)

CNS, central nervous system; RP, relapsing polychondritis.

### Serological examination

The serological examination showed increased serum C-reactive protein (CRP, 90.9%, 10/11) and erythrocyte sedimentation rate (ESR, 81.8%, 9/11) in the vast majority of RP patients. One RP patient was diagnosed with Sjögren's syndrome, presenting increased levels of rheumatoid factor (RF). The remaining was negative for an RF test (Table 1).

### Pronounced signs of inflammation in the CSF of RP patients with CNS involvement

CSF analysis in all RP patients with CNS involvement indicated inflammatory changes, as evidenced by increased levels of neutrophils, total proteins, and immunoglobulin. A slight elevation of CSF cell count(white blood cell, range 6–176/μL) was detected in nine RP patients. The CSF total protein was elevated (>0.45 g/L) in all, with a mean of 1.15 g/L (range 0.51–2.6 g/L); and CSF immunoglobulin G was elevated (>34 mg/L) in all, with a mean of 146.6 mg/L (range 66.8–264 mg/L). Except for one case with unfavorable cooperation, measurements of intracranial pressure (ICP) in the remaining 10 cases demonstrated 5 cases of normal ICP, 2 cases of intracranial hypertension, and 2 cases of intracranial hypotension (Table 1).

### EEG findings in RP patients with CNS involvement

EEG was performed among 10 RP patients with CNS involvement, with only one case presenting normal findings. Generalized abnormalities at a moderate level were the most common abnormal pattern of EEG (*n* = 4), followed by generalized abnormalities at a mild level (*n* = 2). Focal awake EEG abnormalities were detected in one case, manifesting as continuous slow waves at a medium voltage and a frequency of 2.5–5.0 Hz on the left side of the head. Generalized EEG abnormalities at a moderate level combined with focal slowing were observed in another case, showing increased slow waves at a medium-to-high voltage and a frequency of 1.6–5.0 Hz across all leads, especially in the frontopolar lobe on the right side, as well as a small number of sharp and slow waves in the frontal and frontotemporal lobes on the same side (Table 1).

### Pronounced white matter degeneration and brain atrophy on cranial MRI of RP patients with CNS involvement

White matter degeneration (*n* = 7) and brain atrophy (*n* = 7) were the most pronounced changes on the plain cranial MRI (Figure 3). Among the seven cases of atrophy, five presented whole-brain atrophy and two had the shrinkage of the temporal lobes. Interestingly, brain atrophy was found in the vast majority of RP patients with a delayed diagnosis of CNS involvement for more than 2 months (85.7%, 6/7), while one out of the five patients (20.0%, 1/5) with a delayed diagnosis within 2 months was confirmed to have brain atrophy. Additionally, focal parenchymal signal-intensity abnormalities were detected on cranial MRI, including cortical lesions in 4 cases, subcortical lesion in 1 case, and infratentorial lesion in 1 case. Three cases of enhancement at varying degrees were detected out of 6 cases examined with the cranial MRI with contrast, including 1 case of multiple abnormal enhancement patterns on bilateral basal ganglia, thalamus, and brainstem, 1 case of mild and uneven enhancement on bilateral temporo-parieto-occipital (TPO) junctions, and 1 case of leptomeningeal enhancement. Arterial spin labeling (ASL), a non-invasive MRI technique, detected 3 cases of hypoperfusion and 2 cases of hyperperfusion.

**Figure 3:**
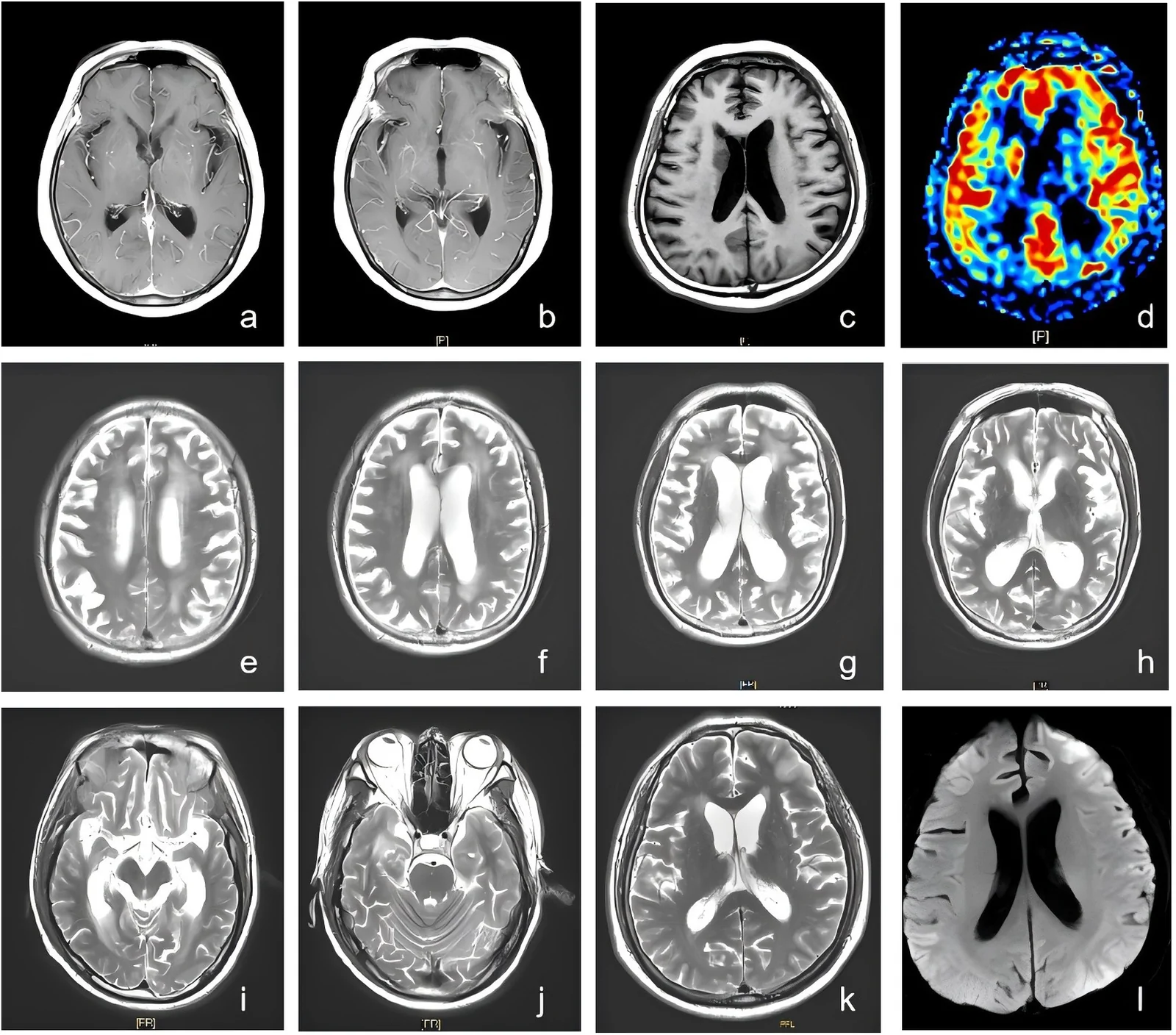
Representative cranial MRI scans of RP patients with CNS involvement. **(a,b)** Contrast-enhanced T1-weighted images of Case 5 indicate leptomeningeal enhancement. **(c,d)** A hypointense T1 signal with a high perfusion in the right corona radiata of Case 3. **(e–h)** A diffuse white matter degeneration combined with brain atrophy of Case 9. **(i,j)** Contrast-enhanced T2 signals in the right temporal lobe, bilateral hippocampi, and white matter of Case 10. **(k,l)** Hyperintense T2 and DWI signals in bilateral frontal lobes of Case 11.

### Vestibular function testing, and audiometry for RP

Decreased vestibulo-ocular reflex (VOR) gains combined with compensatory saccades (CSs) in vestibular function testing were detected in two RP patients with dizziness, suggesting vestibular dysfunction. In addition, hearing loss was reported by four RP patients, and they were finally diagnosed with sensorineural hearing loss (SNHL) by audiometry.

### Treatment and outcomes

All RP patients with CNS involvement were treated with corticosteroids, including 6 cases managed with the methylprednisolone pulse therapy (200 mg to 1,000 mg daily for the first day and a reduced dose after 3–7 days) and 5 cases intervened with low-dose dexamethasone (10 mg/d) or methylprednisolone (50–80 mg/d). Immunosuppressants were additionally given to 6 RP patients, including cyclophosphamide (0.4 g once per week for 2 cases), as well as mycophenolate mofetil (500 mg twice daily, i.e.,), azathioprine (50 mg once daily), leflunomide (10 mg once daily), and hydroxychloroquine (200 mg twice daily), each in one case. Case 6 with the underlying disease of decompensated cirrhosis experienced gastrointestinal bleeding after 3 days of corticosteroid use, warning a treatment discontinuation. Symptoms and signs (e.g., headache, dizziness, cognitive impairment, psychiatric/behavioral symptoms, and limb weakness) were all relieved at varying degrees in the remaining patients. Except for Case 6, RP patients with CNS involvement reported an initial symptom improvement within 5–28 days after the initiation of corticosteroids. However, one case experienced relapse when the dose of corticosteroids decreased to 20 mg daily, and the condition was controlled by one-week methylprednisolone pulse therapy (500 mg daily) (Table 1).

## Discussion

RP causes cartilage matrix destruction and chondrocyte apoptosis, and eventually multi-organ damage. Driven by Th1 immune pathways, RP is characterized by significant increases of serum pro-inflammatory cytokines and chemokines (e.g., IFN-γ and IL-12), forming a complicated inflammation-regulating network (6, 7). At present, no laboratory biomarkers are specific for diagnosing RP, making its diagnosis heavily reliant on physicians' experience in analyzing a combination of clinical manifestations, imaging findings, and laboratory testing. The McAdam and the modified Damiani and Levine criteria are the core criteria for diagnosing RP (8).

Inflammation and deterioration of cartilage are the major clinical manifestations of RP, while CNS involvement is rarely reported in only 3% of patients, but leads to a very high mortality (9, 10). In China, CNS involvement in RP has just an incidence of 13.81% in large-scale studies (11). Neurological manifestations of RP are non-specific, complicated, and diverse. Through literature review of 28 domestic cases of RP with neurological deficits, CNS involvement is the most common feature, including headache, cognitive decline, psychiatric/behavioral symptoms, speech disorder, cranial nerve damage symptoms, consciousness disorder, motor symptoms, ataxia, etc. However, peripheral nerve damage is rarely reported in RP (12, 13). Our study consistently reported cognitive impairment, headache, movement disorders, and epileptic seizures in RP patients with CNS involvement, in which, cognitive impairment and headache were the most common manifestations of neurological deficits (14, 15).

Occurring before, after, or concurrently with CNS damage, extra-CNS symptoms and signs of RP mainly consist of auricular chondritis, arthritis, conjunctivitis, tracheochondritis, vestibulocochlear symptoms, and skin rashes. Auricular swelling with earlobe sparing, a highly specific manifestation of RP, was found in more than 90% of included RP patients. We therefore speculated this as a typical trait of RP with CNS involvement. Cao et al. (12) consistently reported that swelling and inflammation in the ear is more frequently observed in 25 RP patients with CNS involvement than in 156 RP patients without neurologic manifestations.

Encephalitis and ischemic stroke are the most common manifestations of CNS involvement in RP (9, 12). Generally, an immune response is believed to drive neurological deficits in RP. Cerebral and meningeal vasculitis are observed in RP patients with meningitis or meningoencephalitis (16–19). In our study, RP patients were highly suspected to have meningitis or meningoencephalitis. They were negative for pathogens in the next-generation sequencing and specific neural autoantibodies, excluding the cause of autoimmune encephalitis. Moreover, multinucleated cells were found in the CSF of all RP patients, and increased CSF cells and immunoglobulin levels were detected in 90% of RP patients, indicating the arousal of immune-mediated systemic inflammation in RP.

White matter degeneration and brain atrophy were found as the major manifestations on cranial MRI of RP patients. Other imaging features of RP consisted of cortical, subcortical, and infratentorial lesions, as well as meningeal enhancement, suggesting the extensive neurological deficits in the brain region of RP patients. Zhang et al. (10) consistently reported widespread brain lesions in patients with RP-associated meningoencephalitis, including parenchymal lesions and meningeal enhancement in 81% (22/27) and 33% (9/27) of cases, respectively. Wang et al. (19) demonstrated that non-specific white matter changes and a preferential involvement into the medial temporal lobe are typical features on cranial MRI of patients with RP-associated meningoencephalitis. In the present study, one RP patient did not show radiological abnormalities on cranial MRI at the first time of CNS involvement, but presented brain atrophy and abnormal signals in the corona radiata, bilateral basal ganglia, and brainstem during the relapsed episode of CNS involvement at 8 months. We therefore suggested that CNS involvement is progressive in RP, may not synchronize the course of RP. Zhu et al. (20) reported a case of RP with brain lesions expanding increasingly from the bilateral temporal lobes to the white matter. Imaging re-examination confirmed ventricular enlargement and bilateral hippocampal atrophy at 3 months of the pulse steroid therapy, even though white matter lesions gradually resolved. This indicated that immune-mediated inflammation in RP triggers a continuous progression of neurodegeneration (20). Consistently, we found a higher proportion of RP patients with brain atrophy and a course of more than 2 months and white matter degeneration. But the potential relationship between delayed diagnosis and irreversible structural brain changes warrants investigation in larger prospective cohorts. Additionally, ASL measured cerebral blood flow in RP patients with CNS involvement, showing 2 cases of hyperperfusion and abnormal cortical signals without white matter lesions and 3 cases of hypoperfusion in both hemispheres and pronounced white matter lesions. Distinct tissue perfusion in RP with CNS involvement could be attributed to pathophysiological manifestations that altered with the disease progression. In the early active inflammation stage, hyperperfusion in the cortical region is a signal of local vasodilation and hypermetabolism. With the prolongation of CNS involvement in RP, hypoperfusion in the white matter indicates chronic endothelial dysfunction, microvascular damage, or a hypometabolic state secondary to axonal injury (9, 21). Interestingly, different perfusion patterns in the brain may promise as imaging features to distinguish the active inflammation stage from the chronic degeneration stage in RP patients with CNS involvement, offering a novel perspective for illustrating the dynamic evolution of neurological damage in RP.

Corticosteroids are the cornerstone of RP treatment, although unified standards are scant on their dosage, course, and indications for pulse therapies against CNS involvement in RP. Currently, pulse steroid therapy is preferred to RP patients with CNS involvement to prevent irreversible brain lesions in the early stage, and immunosuppressants are added as the second-line treatment to those who are poorly responsive to corticosteroids. Here, the vast majority of RP patients with CNS involvement benefited much from corticosteroids. A systematic review assessed the efficacy of traditional immunosuppressants vs. biological agents in treating RP, concluding that tocilizumab (TCZ) and TNF inhibitors (TNFi) are the drugs associated with the highest response rate, and methotrexate (MTX) presents the most robust outcome of RP treatment. However, rituximab (RTX) and anakinra (ANA) are less effective against RP, and the response of RP patients to azathioprine (AZA) and cyclophosphamide (CYC) fluctuates greatly (22, 23).

Overall, RP is a rare immune-mediated disease with unknown pathogenesis. CNS involvement in RP is even rarer, but with a high mortality, and is difficult to be timely diagnosed and managed due to its non-specific presentations. Neurological deficits, pathologically characterized by both acute inflammation and chronic degeneration in RP, usually stem from immune-mediated vasculitis that can be confirmed by CSF analysis. RP patients with CNS involvement are initially treated with corticosteroids, clutched by additional immunosuppressive or biologic agents. In our cohort of 11 RP patients with CNS involvement, we concluded that auricular swelling with earlobe sparing was the typical clinical manifestation. Their distinct perfusion patterns on cranial MRI further validated the mechanisms involving both acute inflammation and chronic degeneration. Our findings highlighted the importance of CSF analysis and cranial MRI in the early diagnosis and treatment of RP with CNS involvement.

Thus, a notable strength of the present study is the comprehensive diagnostic evaluation performed to exclude alternative causes of the CNS manifestations. Nearly all patients underwent a systematic diagnostic work-up, including CSF analysis, comprehensive testing for autoimmune encephalitis and paraneoplastic antibodies, electroencephalography, and multimodal neuroimaging. Strict exclusion criteria were applied to rule out CNS infection, autoimmune encephalitis, acute ischemic stroke, and other structural or neurodegenerative lesions. This systematic and multidisciplinary approach to differential diagnosis, together with the consecutive enrollment of patients and the blinded independent review of imaging findings, substantially strengthens the attribution of the observed CNS involvement to RP. We believe that the rigor of this diagnostic work-up supports the validity of our observations and provides a useful reference for the recognition and evaluation of CNS involvement in RP.

### Limitations

Among the several limitations of this study, the uncertainty regarding the nature and attribution of the CNS involvement merits particular attention. Although we performed a comprehensive diagnostic evaluation to exclude alternative causes—including CNS infection, autoimmune encephalitis, acute infarction, and other structural or neurodegenerative lesions—the relatively advanced age of our cohort (mean 62.7 ± 9.3 years) and the presence of vascular risk factors in 7 of 11 patients (63.6%) raise the possibility that the observed white matter degeneration and brain atrophy partly reflect age-related or vascular changes rather than RP-specific CNS involvement. White matter hyperintensities are common in asymptomatic elderly individuals, are strongly associated with vascular risk factors and cerebral small vessel disease (24), and show largely overlapping regional distributions across vascular dementia, Alzheimer's disease, and healthy aging (25). Conversely, CNS involvement in RP is rare and frequently accompanied by non-specific or even normal MRI findings and can be easily misdiagnosed as infection or other inflammatory disorders (26). Although alternative etiologies were carefully excluded, concurrent or prodromal neurodegenerative pathology, particularly in patients with longstanding vascular risk factors, cannot be fully excluded. MRI findings should therefore be interpreted together with the clinical picture, CSF parameters, and response to immunosuppressive therapy, and the attribution of these changes specifically to RP-CNS involvement warrants caution. Prospective studies with longitudinal imaging, quantitative measures, and neuropsychological assessment are required to distinguish RP-related injury from age-related and neurodegenerative changes. Therefore, we believe that the present study has the following deficiencies or limitations. (i) the retrospective, single-center design and potential selection bias; (ii) the uncertainty in attributing the observed CNS manifestations and imaging changes specifically to RP, given the advanced age and the presence of vascular risk factors in part of the cohort; (iii) the small sample size (*n* = 11), which limits the statistical power and generalizability; (iv) the absence of a control group; (v) the lack of systematic long-term follow-up; (vi) the non-uniform neuroimaging protocol (not all patients underwent contrast-enhanced MRI or ASL); and (vii) missing data inherent to the retrospective design. In addition, future prospective studies with systematic OCB testing and intrathecal IgG synthesis analysis are warranted to further characterize the immunopathogenesis of CNS involvement in RP.

## Data Availability

The original contributions presented in the study are included in the article/Supplementary Material, further inquiries can be directed to the corresponding authors.
